# PLK1 Interacts and Phosphorylates Axin That Is Essential for Proper Centrosome Formation

**DOI:** 10.1371/journal.pone.0049184

**Published:** 2012-11-14

**Authors:** Ka Ruan, Fan Ye, Chenyu Li, Yih-Cherng Liou, Sheng-Cai Lin, Shu-Yong Lin

**Affiliations:** 1 State Key Laboratory of Cellular Stress Biology, School of Life Sciences, Xiamen University, Xiamen, Fujian, China; 2 Department of Biological Science, National University of Singapore, Singapore, Republic of Singapore; Cedars-Sinai Medical Center, United States of America

## Abstract

Abnormal amplification of centrosomes could lead to improper chromosome segregation and aneuploidy and is implicated in cancer development. Here, we demonstrate that Axin, a scaffolding protein in Wnt signaling, is phosphorylated by PLK1 during mitosis. Phosphorylation of Axin Ser-157 by PLK1 abolished Axin association with γ-tubulin, while substitution of Ser-157 with alanine exhibited sustained interaction with γ-tubulin. In addition, overexpression of Axin-S157A significantly increased the number of cells with multi-centrosomes. These results suggest that the phosphorylation status of Axin, mediated by PLK1, dynamically regulates its association with γ-tubulin and centrosome formation and segregation.

## Introduction

Centrosome defects are implicated as a primary cause of chromosomal instability and aneuploidy in cancer development [1]–[3]. Abnormal increase in the number of centrosome can adversely affect spindle function and cytokinesis, leading to the formation of multi-polar spindles that promote chromosome mis-segregation and polyploidy, and ultimately genetic instability [4], [5]. Polo-like kinases (PLK) play key roles during multiple stages of mitosis, from prophase to cytokinesis [6], [7]. Endogenous protein levels of PLK1 are up-regulated during late G2 and M phases, where it appears to regulate the mitotic processes by phosphorylating a series of substrates including Cdc25C and ROCK2 [8]–[10]. Overexpression of PLK1 in tumors is frequently associated with DNA aneuploidy and centrosome amplification. Conversely, knockdown of *PLK1* by small interference RNAs (siRNAs) suppresses centrosome amplification [11], [12].

Axin, a negative regulator of Wnt signaling, is a versatile scaffold protein, involved in multiple signaling pathways including the JNK MAP kinase cascade, and p53 signaling in DNA damage response [13]–[15]. Moreover, Axin is found to be present on the centrosome and along the mitotic spindle, which is regulated by Aurora kinases [16]. The co-localization between Axin and centrosome was also identified in *Xenopus* and this localization requires the c-terminal region of Axin that includes a DIX domain [17]. In addition, Axin2, another member of the Axin protein family, was also shown to co-localize with the mitotic spindles of colon cancer cells [18]. The centrosomal localization of Axin is likely dependent on the association between Axin and γ-tubulin as knockdown of *Axin* affects the proper localization of γ-tubulin to the centrosome and compromises the centrosomal microtubule nucleation activity after nocodazole treatment [19]. On the other hand, overexpression of Axin facilitates multinuclear giant cell formation in the gastric cancer cell line AGS [20]. Ectopically expressed Axin alters the distribution of PLK1 and GSK3β, and renders the cells able to bypass the cytokinesis failure mediated by Aurora inhibition through an aberrant actin-based cytokinesis [16].

In this study, we identify that Axin is a direct phosphorylation substrate of PLK1. The critical residue for PLK1 phosphorylation was mapped to residue Ser-157 of mouse Axin, phosphorylation of which leads to dissociation between Axin and γ-tubulin. In addition, we also demonstrate that ectopic expression of Axin S157A mutant results in sustained interaction of Axin and γ-tubulin, and leads to abnormal multi-centrosome formation. Our study thus provides strong evidence for the link between Axin and centrosome formation, and will shed new light on the causes of tumorigenesis resulting from dysregulated mitosis.

## Results

### 1. Phosphorylation of Axin by PLK1

**Figure 1:**
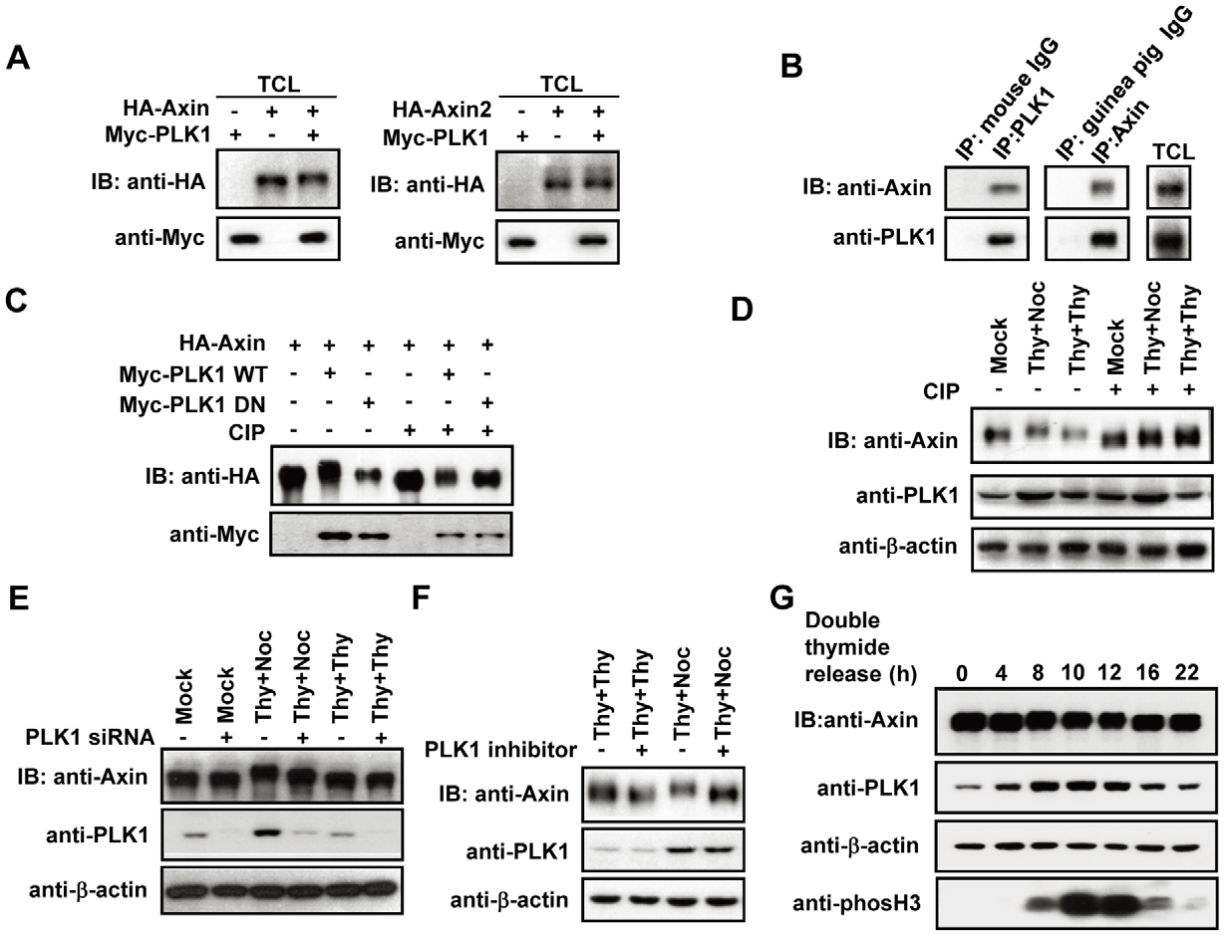
Axin phosphorylation by PLK1. (A) Total cell lysates (TCL) from cells expressing Axin or Axin2 with PLK1 or empty vector in HEK293T cells were analyzed by immunoblotting as indicated. (B) Axin interacts with PLK1 at its endogenous level. The HeLa cell lysates were immunoprecipitated with anti-Axin, mouse anti-PLK1 or control IgGs, respectively, followed by immunoblotting as indicated. (C) HA-Axin immunoprecipitates were subjected to CIP treatment and immunoblotting as indicated. (D) HeLa cells were mock treated or arrested in M-phase or early-S-phase by treatment with double-thymidine or thymidine-nocodazole. Axin immunoprecipitates were treated with CIP, followed by immunoblotting. (E, F) TCLs from cells expressing siRNA against *PLK1* (E) or were treated with 50 nM of PLK1 inhibitor 10 h before harvest (F) were analyzed by immunoblotting as indicated. (G) PLK1 phosphorylation on Axin at G2/M transition. HeLa cells were synchronized by double-thymidine block and released in fresh medium for indicated times. TCLs were analyzed by western blot and probed with antibodies as indicated.

Axin is reported to localize at the mitotic spindle throughout the mitotic phase when PLK1 kinase activity accumulates to maximum during the cell cycle. We found that both Axin and Axin2 interact with PLK1 (Figure 1A, B) [16], [18]. When exploring biochemical consequence of the interaction between PLK1 and Axin, we found that Axin displayed an up-shift in mobility on the SDS-PAGE gel when co-expressed with PLK1, whereas Axin2 did not show such a mobility shift (Figure 1A). We then immunoprecipitated Axin from cells co-expressing both Axin and PLK1 and subjected the immunoprecipitates to treatment with or without calf intestinal phosphatase (CIP). In the untreated group, Axin co-expressed with wild-type (WT) PLK1 but not kinase dead K82M mutant (DN) PLK1 had a slower mobility on the gel, compared to that co-expressed with a control vector. After CIP treatment, the mobility difference between the three samples was abolished (Figure 1C), indicating that the mobility shift of Axin is caused by phosphorylation. As PLK1 is predominantly expressed in the late-G2 and M-phase [6], HeLa cells were synchronized and arrested in the M-phase with thymidine-nocodazole treatment to elevate PLK1 protein levels or in the early-S-phase with double-thymidine treatment as a control. An up-shift in mobility of the immunoprecipitated endogenous Axin from the M-phase cells compared to the untreated or early-S-phase cells was observed, which disappeared after the CIP treatment (Figure 1D). Either knockdown of *PLK1* by siRNA or treatment with PLK1 inhibitor abolished the mobility shift of Axin in the M-phase arrested cells without affecting Axin in untreated or the early-S-phase arrested cells (Figure 1E, F). Up-shift of Axin was also observed in HeLa cells that progressed synchronously from early-S-phase to the next G1-phase (Figure 1G), indicating that PLK1 is the kinase that phosphorylates Axin during M-phase.

### 2. Identification of PLK1 phosphorylation sites on Axin

**Figure 2:**
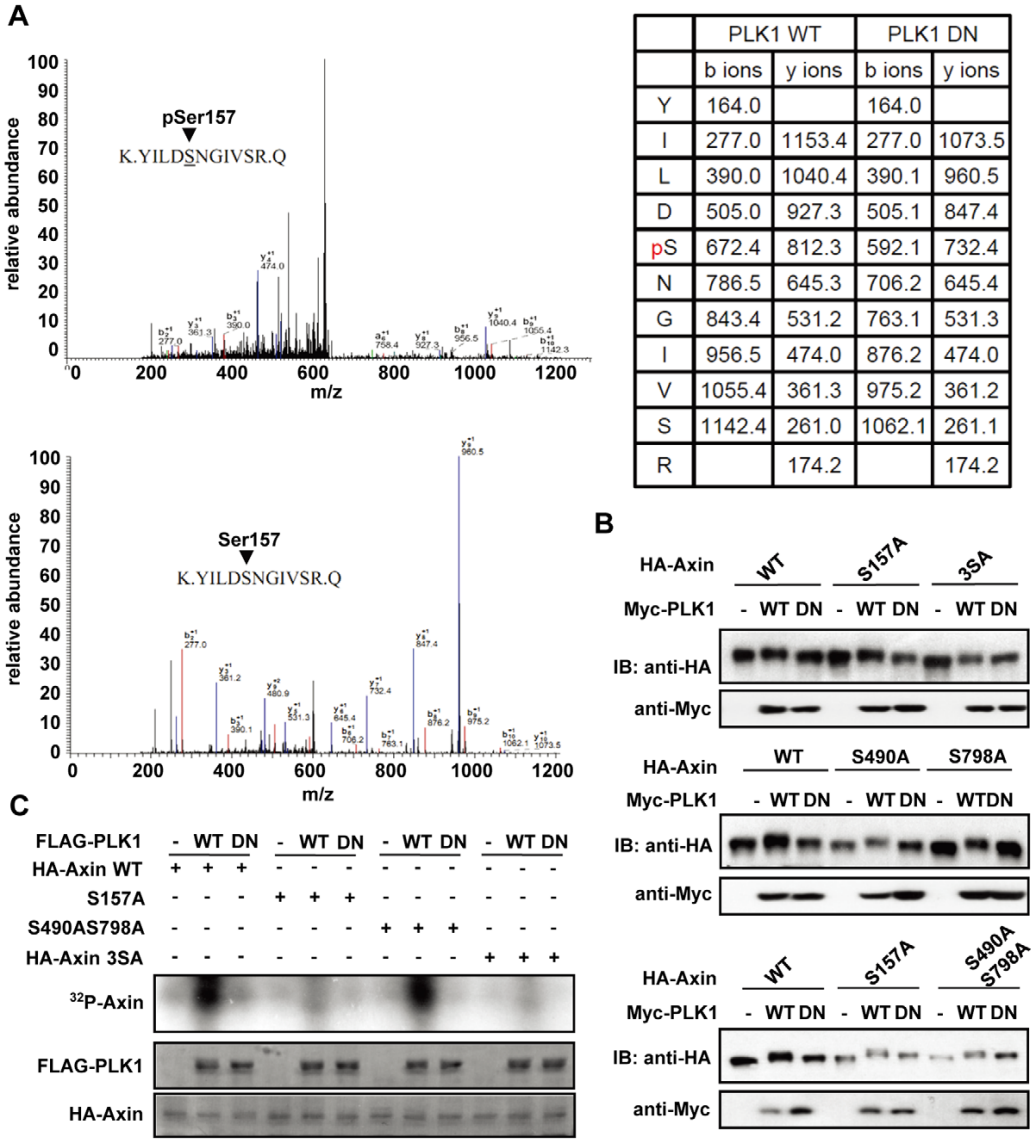
Identification of PLK1 phosphorylation sites on Axin. (A) Mass spectrometry analysis using Axin immunoprecipitates shows that Ser-157 in Axin is one of the phosphorylation sites of PLK1. Upper panel: HA-Axin was immunoprecipitated from cells co-expressing PLK1-WT; lower panel: from cells co-expressing PLK1-DN. (B) Axin-3SA mutant no longer displays its mobility shift when co-expressed with PLK1. HA-tagged Axin-WT, Axin-S157A, Axin-3SA, Axin-S490A, and Axin-S798A, Axin-S490A/S798A mutants are co-expressed with Myc-PLK1-WT or Myc-PLK1-DN in HEK293T cells, respectively. At 24 h post-transfection, cell lysates were subjected to immunoblotting as indicated. (C) Phosphorylation of Axin by PLK1 *in vitro*. HEK293T cells were transfected with HA-Axin-WT or HA-Axin serine-to-alanine mutants, and cell extracts were immunoprecipitated with HA-antibody. The immunocomplexes were incubated with purified FLAG-PLK-WT or FLAG-PLK1-DN in the presence of [γ-^32^P] ATP for the kinase assay. Following the assay, samples were subjected to Coomassie Blue staining and autoradiography as indicated.

In order to locate the exact amino acid residue(s) of Axin that is phosphorylated by PLK1, we utilized mass spectrometric analysis to examine HA-Axin samples immunoprecipitated from HEK293T cells co-expressing PLK1-WT or PLK1-DN. The residue Ser-157 of Axin was found to be phosphorylated only in cells co-expressing PLK1-WT but not PLK1-DN (Figure 2A). However, S157A mutant retained mobility shift when co-expressed with PLK1 (Figure 2B). We then continued to substitute a series of other potentially phosphorylated serine or threonine residues with alanine. When Ser-157, Ser-490 and Ser-798 (Axin-3SA) were simultaneously mutated, the mobility up-shift no longer occurred, while single mutations of Ser-490 and Ser-798 or double mutation of these two sites did not abrogate the mobility shift (Figure 2B). An *in vitro* kinase assay using wild-type or serine-to-alanine mutants of Axin as substrates for PLK1 was performed to test if these sites are direct PLK1 phosphorylation sites. As shown in Figure 2C, Axin mutants with Ser-157 altered abolished *in vitro* phosphorylation by PLK1, but S490A and S798A mutations have no such an effect. These results suggest that only Ser-157 is the direct phosphorylation site for PLK1, while Ser-490 and Ser-798 are not directly phosphorylated by PLK1 but through other PLK1 kinase activity dependent events.

### 3. Phosphorylation status of Axin determines its affinity for γ-tubulin

**Figure 3:**
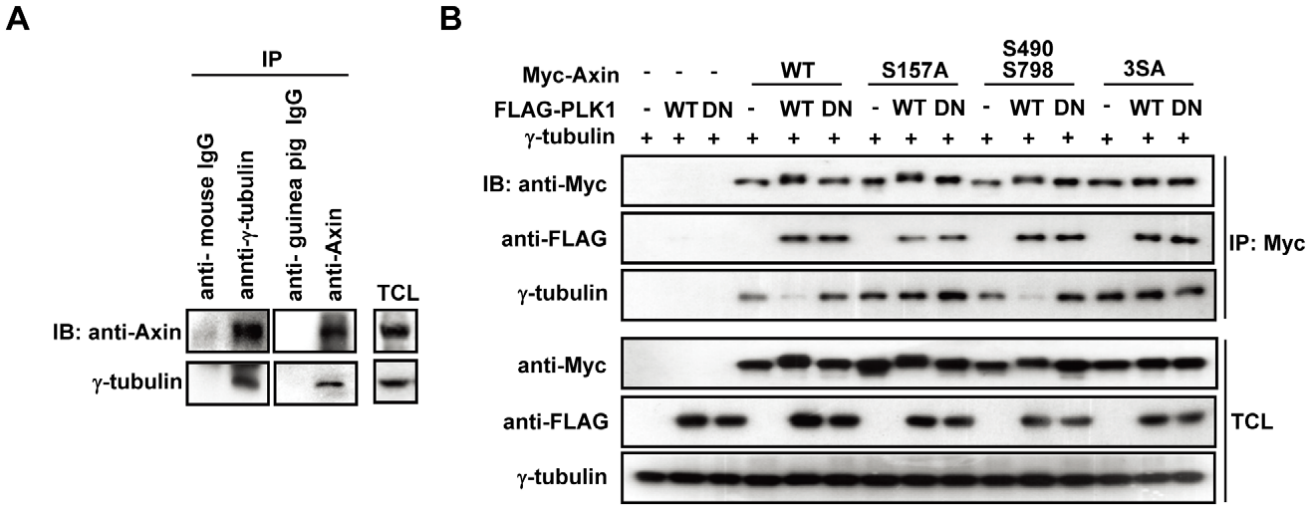
Axin phosphorylation by PLK1 regulates Axin-γ-tubulin association. (A) Axin interacts with γ-tubulin at its endogenous level. (B) Axin-S157A and Axin-3SA show a sustained interaction with γ-tubulin. HEK293T cells were transfected as indicated. TCLs and Axin immunoprecipitates were subjected to immunoblotting as indicated.

It was previously reported that Axin but not Axin2 can form a complex with γ-tubulin [19]. Coincidently, we also found that Axin but not Axin2 shows mobility shift when co-expressed with PLK1 (Figure 1A). To test the possibility that the selective phosphorylation on Axin may have a role in regulating Axin-γ-tubulin interaction, we first verified interaction between endogenous Axin and γ-tubulin in HEK293T cells (Figure 3A). Co-expression of PLK1-WT but not PLK1-DN reduced the amount of γ-tubulin co-immunoprecipitated with Axin, while Axin-S157A and Axin-3SA showed a sustained interaction between Axin and γ-tubulin, neglecting the effect caused by co-expression of PLK1-WT (Figure 3B). Consistent with our conclusion that Ser-490 and Ser-798 are not directly phosphorylated by PLK1 which is drawn from the *in vitro* kinase assay, the Myc-Axin-S490/798A double-point mutant behaved similarly to the wild-type Axin in γ-tubulin binding (Figure 3B). These results suggest that Ser-157 phosphorylation of Axin by PLK1 alone is sufficient to regulate Axin-γ-tubulin association.

**Figure 4:**
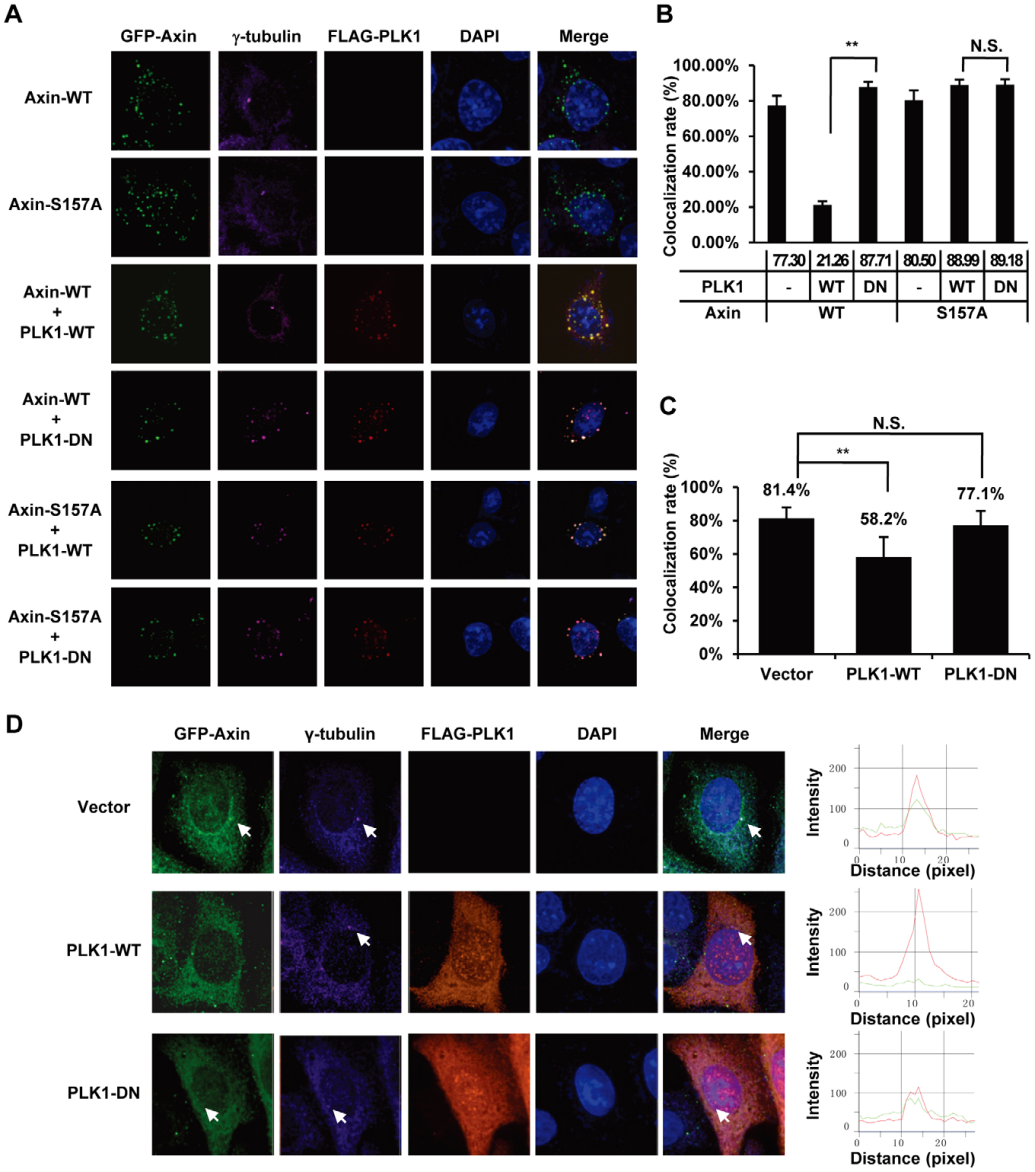
Co-expression of PLK1 abolishes Axin-γ-tubulin co-localization. (A) GFP-Axin-WT or GFP-Axin-S157A was co-transfected with empty vector, FLAG-PLK1-WT or FLAG-PLK1-DN into HeLa cells. Cells were fixed and immuno-stained as described in Materials and Methods. Axin was visualized by GFP, PLK1 by using anti-FLAG (red), γ-tubulin by using anti-γ-tubulin (pink), and nuclei by DAPI staining. (B) Bar chart showing the quantification data for the co-localization rates between γ-tubulin and GFP-Axin-WT or GFP-Axin-S157A at γ-tubulin foci in the background of FLAG-PLK1 or FLAG-PLK1-DN overexpression as shown in (A). *n* = 30 (“*n*” indicates the number of the cells quantified), N.S.: not significant; **P<0.01 (ANOVA followed by tukey). (C) The centrosome region was circled and analyzed using Volocity software. The co-localization rate was analyzed between 488 channel (Axin) and 642 channel (γ-tubulin). Statistical analysis was performed as in (B). (D) FLAG-PLK1-WT and FLAG-PLK1-DN were transfected respectively into HeLa cells. Goat anti-Axin, mouse anti-FLAG and rabbit anti-γ-tubulin were used for staining of endogenous Axin, PLK1 and γ-tubulin, respectively. The intensity of endogenous Axin and γ-tubulin staining along the γ-tubulin foci is shown by line profiles. A 2 µm line is drawn along the centrosome region and the Y axis shows the intensity of 488 channel (Axin) and 642 channel (γ-tubulin) along the distance of the line (X axis).

The subcellular localization of Axin, PLK1 and γ-tubulin was also investigated by immunostaining in HeLa cells (Figure 4A and Figure S1). Consistent with the co-immunoprecipitation results, the co-localization rate between GFP-Axin and endogenous γ-tubulin at γ-tubulin positive foci was greatly compromised by co-expression of PLK1-WT but not PLK1-DN (21.26% vs. 87.71%, Figure 4B and Figure S2), while PLK1-WT co-expression did not disrupt the co-localization between the endogenous γ-tubulin and the GFP-Axin-S157A mutant. We further investigated the effect of PLK1 overexpression on centrosome localization of endogenous Axin. As shown in Figure 4C and D, PLK1-WT reduced Axin-γ-tubulin co-localization rate at the centrosome region to 58.2% compared to that in control cells (81.4%) or PLK1-DN expressing cells (77.1%), suggesting a PLK1 kinase activity dependent regulation of Axin and γ-tubulin association and co-localization.

### 4. Axin Ser-157 mutation causes multi-centrosome formation

**Figure 5:**
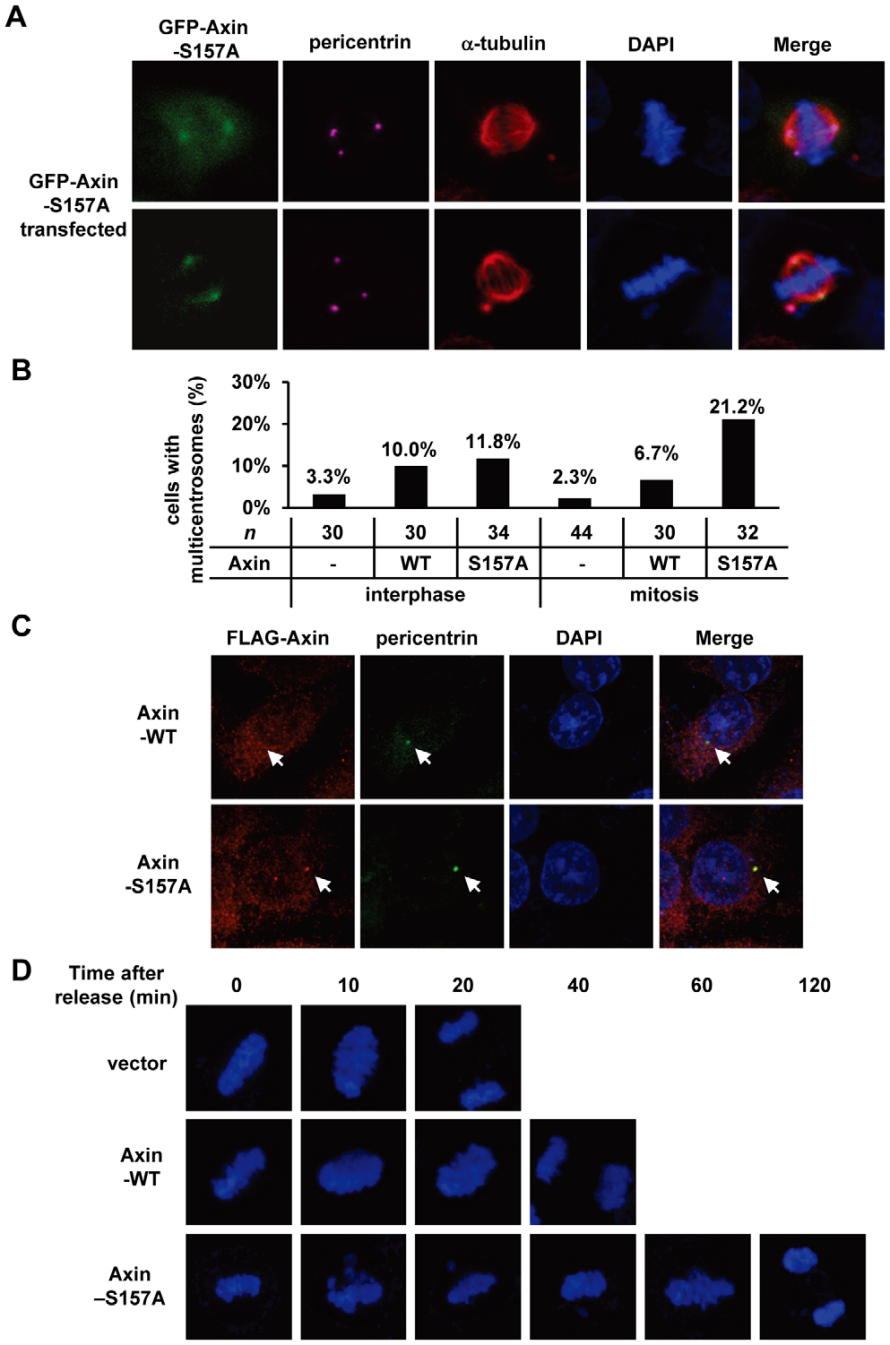
Altered centrosome number in Axin-S157A expressing mitotic cells. (A) HeLa cells were transfected with GFP-Axin-S157A. Cells were treated with thymidine-nocodazole, then fixed and stained as described in Materials and Methods. GFP staining for Axin, anti-α-tubulin staining for microtubule (red), anti-pericentrin staining for centrosome (pink), and nuclear staining by DAPI were performed. (B) Bar chart representing the percentage of the interphase or mitotic HeLa cells with multiple centrosomes. Cells were transfected with GFP vector (control), GFP-Axin-WT or GFP-Axin-S157A mutant. “*n*” indicates the number of the mitotic cells quantified. The differences among interphase groups are not significant, *P* = 0.528; the differences among mitosis groups are significant (*P* = 0.014, Vector^a^; Axin-WT^a,b^; Axin-S157A^b^, Chi-Square Tests). (C) Centrosome numbers are not altered in Axin-WT or Axin-S157A expressing interphase cells. HeLa cells were transfected with FLAG-Axin-WT or FLAG-Axin-S157A. Axin was visualized by using anti-FLAG (red), pericentrin by anti-pericentrin (green), and nuclei by DAPI staining. (D) Axin-S157A overexpression delays the chromosome segregation. The cells were synchronized at metaphase and released in fresh medium followed by staining with DAPI.

To explore functional significance of Axin phosphorylation by PLK1, we transfected GFP-Axin-S157A into HeLa cells. We found that overexpression of Axin-S157A mutant led to an increased ratio of multi-centrosome containing mitotic cells from 2.3% (control) to 21.2% (Axin-S157A transfected) as indicated by the centrosome marker pericentrin (Figure 5A, B). We also quantified the percentage of multi-centrosome-containing mitotic cells in the background of GFP-Axin-WT overexpression. The results show that overexpression of GFP-Axin-WT induced only 6.3% of mitotic cells with multi-centrosome, indicating that the PLK1 phosphorylation of Axin is important for proper centrosome duplication.

As shown in Figure 4A, multiple γ-tubulin foci were observed in Axin and PLK1 coexpressing cells. To investigate whether these foci are extra-centrosomes, we examined centrosome numbers in interphase cells overexpressing Axin or its S157A mutant. Consistent with the observation that Axin are largely unphosphorylated by PLK1 in non-mitotic cells (Figure 1, E to G), the centrosome numbers in interphase cells are not significantly altered by Axin-S157A overexpression (Figure 5B, C). Similar to Axin-WT expressing mitotic cells, a moderate increase of pericentrin positive foci was observed in Axin-WT (10.0%) or Axin-S157A (11.8%) expressing interphase cells. These extra-centrosomes could be partly derived from the mitotic cells with altered centrosome numbers. To investigate the effect of multi-centrosomes on the process of mitosis, HeLa cells were synchronized at metaphase and released at different time points. Chromosomes began to segregate in 20 min in control cells, whereas chromosome segregation in cells overexpressing Axin-S157A was postponed to about 2 h after release, which indicates that the multi-centrosome phenotype leads to partial block of mitosis (Figure 5D).

## Discussion

In the present study, we have provided evidence to indicate that Axin is a substrate for the protein kinase PLK1 that localizes to centrosome and is required for proper chromosome separation during mitosis. Combined results from mass spectrometric analysis and mutational studies revealed that Ser-157 of Axin is directly phosphorylated by PLK1. Through co-immunoprecipitation experiments, we found that γ-tubulin preferentially associates with unphosphorylated Axin. Importantly, mutation of Ser-157 on Axin caused a significant increase of cells with multi-centrosomes, consistent with a role of PLK1 in proper centrosome formation and chromosome segregation [21].

Same as DNA replication, centrosomes are licensed to duplicate once per cell cycle to ensure genetic stability. The exact mechanism for centrosome amplification in tumor cells is not clearly understood, but extra-centrosomes could result from defects in the control of the centrosome replication cycle, failure in cytokinesis or cell fusion. In our study, the appearance of extra-centrosomes is more obvious in mitotic cells than in interphase cells, indicating that altered centrosome replication cycle could be the primary cause of multi-centrosome formation. The post-translational modifications of the pericentriolar component γ-tubulin have been reported to be critically important for the proper duplication of the centrosomes and the maintenance of an appropriate centrosome number in the cell. γ-tubulin should be dynamically ubiquitinated and phosphorylated during centrosome cycle. Specifically, non-ubiquitinable or phosphomimetic γ-tubulin mutants increase centrosome numbers, whereas un-phosphorylatable γ-tubulin impaired centrosome duplication [22], [23]. In view of the dynamic phosphorylation of Axin, it would be expected that the ubiquitination or phosphorylation status of γ-tubulin is regulated by Axin through its Ser-157 phosphorylation- status-dependent interaction between these two proteins.

Correct number of centrosomes is critical for proper chromosome segregation during cell division and for the prevention of aneuploidy, a hallmark of cancer [1]–[3]. However, recent studies reveal a direct link between increased centrosome number and cancer, in which extensive aneuploidy did not occur [24], [25], indicating that centrosome amplification itself may be sufficient to promote tumor formation. In our study, the extra-centrosomes observed in GFP-Axin-S157A transfected HeLa cells do not possess the same microtubule nucleating ability, and the spindle remained bi-polar. Hence these cells are not expected to become aneuploid. However, these extra-centrosomes are not simply innocent bystanders, whose appearance associates with a delay in chromosome segregation in these cells (Figure 5D) and could lead to other yet uncharacterized defects such as disruption of cell polarity, interfering with normal cell behavior and tissue architecture. The roles of these extra-centrosomes as well as the fate of the cells with multi-centrosomes require further investigation and could provide insight into a new mechanism for tumor formation and cancer development. It is intriguing to note that another component in Wnt signaling, Dvl2, is also phosphorylated by PLK1 [26]. Together with the report that PLK1 is overexpressed in colorectal cancers, the majority of which are characterized by dysregulated Wnt signalling [27], it is of interest to test whether PLK1 plays a role in regulating Wnt/β-catenin signalling in the future.

## Materials and Methods

### 1. Plasmid constructions

Expression vectors for HA- or Myc-tagged wild-type mouse Axin were constructed as previously described [14]. For the construction of GFP-tagged wild-type Axin or its serine-to-alanine mutants, wild type and mutants of Axin were digested internally at NdeI and SmaI sites from pCMV5-HA-Axin, blunted with Klenow fragment of pol I (Takara), gel purified, and inserted into a SmaI digest of GFP-C1 vector. HA-, Myc-, FLAG-PLK1 were generated by PCR amplification. PLK1 kinase-dead mutation was created by site-directed mutagenesis using the Transformer™ site-directed mutagenesis kit (CLONTECH). γ-tubulin was amplified from a human brain cDNA. All PCR products were verified by sequencing. Sequences for all the oligonucleotides used in this study are available upon request.

### 2. Cell culture, synchronization and transient transfection

HEK293T and HeLa cells were cultured in Dulbecco's modified Eagle's medium (DMEM) supplemented with 10% fetal bovine serum (FBS). To obtain M-phase arrested cells, HeLa cells were incubated with the medium containing 2 mM thymidine for 24 h. The cells were then cultured with fresh medium for 3 h, followed by incubation in fresh medium containing 200 ng/ml nocodazole for additional 12 h. To arrest the cells in early-S-phase, HeLa cells were treated with 2 mM thymidine for 18 h, and were then changed to fresh medium for 9 h, followed by a second treatment in the medium containing 2 mM thymidine for 17 h. Transfection was performed using Polyethylenimine.

### 3. Antibodies

Mouse anti-HA (F-7), anti-Myc (9E10) and goat anti-Axin1 (S-20) were purchased from Santa Cruz Biotechnology. Mouse anti-FLAG (M2), mouse anti-β-actin, mouse anti-α-tubulin, rabbit anti-pericentrin and mouse anti-γ-tubulin were purchased from Sigma. Mouse anti-PLK1 (DR1037) was purchased from Calbiochem. Rabbit anti-FLAG was purchased from Cell Signaling Technology. Rabbit anti-phosH3 was purchased from Abcam. The polyclonal antibody against Axin (C2b) was described previously [15].

### 4. CIP assay

In brief, Axin proteins were immunoprecipitated, washed with lysis buffer twice and then the phosphatase buffer twice. The immunoprecipitates were then treated with calf intestinal phosphatase (CIP) in the assay buffer at 37°C for 30 min and the reaction was stopped with 2×SDS sample buffer.

### 5. *In vitro* phosphorylation of Axin

Kinase (wild-type and kinase-dead PLK1) and substrates (Axin proteins) were purified from HEK293T cells. 500 µg of protein lysates from HEK293T cells at 24 h post-transefection was used for immunoprecipitation of FLAG-PLK1 or HA-Axin for 4 h with FLAG or HA antibodies separately. The immunoprecipitates were washed three times with lysis buffer and twice with kinase buffer, and the FLAG-PLK1 was then eluted with 0.2 mg/ml M2 FLAG peptide (Sigma-Aldrich). To start the kinase reaction, kinase and substrate were incubated in 20 µl of kinase buffer (25 mM Tris-HCl, pH 7.5, 150 mM NaCl, 10 mM MgCl_2_, 2 mM dithiothreitol, 5 mM β-glycerophosphate) supplemented with 50 µM ATP, 5 µCi of [γ-^32^P] ATP incubated at 30°C for 30 min. Reactions were terminated by adding an equal volume of 2×SDS sample buffer. The samples were separated on SDS-PAGE and analyzed by Coomassie Blue staining and then dried prior to autoradiography.

### 6. PLK1 siRNA

The N19 sequence targeting PLK1 is: 5′-GGGCGGCTTTGCCAAGTG-3′. Knockdown of *PLK1* was mediated by lentivirus infection system. Briefly, the virus carrying pLL3.7-*PLK1* was prepared by transfecting HEK293T cells with pLL3.7-*PLK1*, pVSVG, pMDL and pREV. 24 h post-transfection, medium containing virus was collected and added into the culture medium of HeLa cells. At 24 h post-infection, HeLa cells were subcultured and kept in DMEM containing 100 mg/ml G418.

### 7. Immunofluorescence staining and imaging analyses

HeLa cells grown on coverslips in 6-well plates were transfected with indicated plasmids. After 24 h, cells were washed with PBS twice, fixed in methanol for 10 min at −20°C, and incubated in 0.5% Triton-X-100 for 10 min followed by blocking in 5% BSA for 1 h. Cells were then incubated for 1 h with primary antibodies at room temperature. After being washed five times with PBS, cells were incubated with Texas Red-conjugated goat anti-rabbit IgG or Cy5-conjugated goat anti-mouse IgG for 1 h. Cells were visualized under a Zeiss 510 META fluorescence confocal microscope (Zeiss).

The line profile was generated using Image pro-plus 6 software. A 2 µm line was draw along centrosome region. The intensities of 488 channel and 642 channel were shown in the graph. The X axis indicates the localization along the line. The Y axis indicates the localization pattern of the two channels—488 channel (Axin) and 642 channel (γ-tubulin).

To analyze co-localization rate of Axin and γ-tubulin, the γ-tubulin positive foci were circled and analyzed using Volocity software.

## Supporting Information

Figure S1 **GFP protein did not colocalize with γ-tubulin.**
(DOC)

Figure S2 **Colocalization of GFP-Axin and γ-tubulin in interphase cells.**
(DOC)
