# Supplementary material for: PLK1 Interacts and Phosphorylates Axin That Is Essential for Proper Centrosome Formation
Source: PLoS One. 2012 Nov 14;7(11):e49184. doi: 10.1371/journal.pone.0049184 (PMC3498349; doi:10.1371/journal.pone.0049184)
Supplement: Figure S1 — GFP protein did not colocalize with γ-tubulin. (DOC) [file pone.0049184.s001.doc]

**Figure S1**

**GFP protein did not colocalize with γ-tubulin.** HeLa cells were transfected with GFP vector as a control and FLAG-PLK1-WT or FLAG-PLK1-DN. The cells were fixed with methanol at -20 °C for 10 min. Rabbit anti-γ-tubulin and mouse anti-FLAG antibodies were utilized.
