# Supplementary material for: PLK1 Interacts and Phosphorylates Axin That Is Essential for Proper Centrosome Formation
Source: PLoS One. 2012 Nov 14;7(11):e49184. doi: 10.1371/journal.pone.0049184 (PMC3498349; doi:10.1371/journal.pone.0049184)
Supplement: Figure S2 — Colocalization of GFP-Axin and γ-tubulin in interphase cells. (DOC) [file pone.0049184.s002.doc]

**Figure S2**

**Colocalization of GFP-Axin and γ-tubulin in interphase cells.** HeLa cells were transfected with empty vector and GFP-Axin or GFP-Axin-S157A. The line profiles were generated using image pro-plus 6 software. A 2 μm line was drawn along centrosome region. The relative intensities of 488 channel and 642 channel were shown in the graph. The X axis indicates the localization along the line. The Y axis indicates the localization pattern of the two channels-488 channel (Axin) and 642 channel (γ-tubulin). The line profiles demonstrate colocalization between Axin and γ-tubulin.
